# Percutaneous Versus Surgical Cannulation for Femoro‐Femoral Venoarterial Extracorporeal Membrane Oxygenation: A Retrospective Cohort Study on Cannulation‐Related Complications

**DOI:** 10.1111/aor.70061

**Published:** 2025-11-21

**Authors:** Axel Dimberg, Magnus Dalén, Anders Franco‐Cereceda, Thomas Fux

**Affiliations:** ^1^ Department of Molecular Medicine and Surgery Karolinska Institutet Stockholm Sweden; ^2^ Department of Cardiothoracic Surgery Karolinska University Hospital Stockholm Sweden; ^3^ Department of Physiology and Pharmacology Karolinska Institutet Stockholm Sweden; ^4^ Department of Perioperative Medicine and Intensive Care Karolinska University Hospital Stockholm Sweden

**Keywords:** cannulation‐related complications, femoral cannulation, percutaneous cannulation, surgical cannulation, V‐A ECMO, venoarterial ECMO

## Abstract

**Background:**

Cannulation for peripheral veno‐arterial extracorporeal membrane oxygenation (V‐A ECMO) can lead to severe local complications. This study evaluated site‐specific complications between percutaneous and surgical cannulation techniques for femoro‐femoral V‐A ECMO, focusing on site bleeding, infection, and limb ischemia.

**Methods:**

We conducted a single‐center retrospective cohort study of adult patients who received femoro‐femoral V‐A ECMO either by percutaneous or surgical technique at a tertiary center. Cannulation‐site complications were assessed from cannulation to post‐decannulation. Risk factors for site bleeding, infection, and limb ischemia within 90 days of cannulation were analyzed using logistic regression.

**Results:**

Among 384 patients (22.4% female), 181 (47.1%) underwent percutaneous, and 203 (52.9%) underwent surgical cannulation. Percutaneous cannulation was associated with significantly fewer patients experiencing site bleeding (29.3% vs. 40.9%, *p* = 0.02) and infection (8.3% vs. 31.0%, *p* < 0.001), with no significant difference in limb ischemia (11.6% vs. 15.3%, *p* = 0.29). 90‐day survival was similar between groups (43.6% vs. 49.8%, *p* = 0.81). Multivariable logistic regression identified surgical cannulation as an independent risk factor for site bleeding (OR 2.39, 95% CI 1.43–3.98; *p* < 0.001) and infection (OR 5.47; 95% CI 2.47–12.12; *p* < 0.001). Limb ischemia was not significantly associated with the cannulation technique but with two other modifiable factors at cannulation: absence of distal perfusion catheterization and larger arterial cannula size.

**Conclusion:**

Percutaneous cannulation was associated with significantly fewer patients experiencing site bleeding and infection compared to surgical cannulation. Limb ischemia was not associated with the cannulation technique but was influenced by two other modifiable factors at cannulation: distal perfusion catheterization and arterial cannula size.

## Graphical abstract

Percutaneous femoro‐femoral V‐A ECMO cannulation was associated with more than a two‐fold lower risk of site bleeding and nearly a six‐fold lower risk of infection compared with surgical cut‐down, with no difference in limb ischemia. Absence of distal perfusion catheterization and larger arterial cannula size were additional modifiable ischemia risk factors.

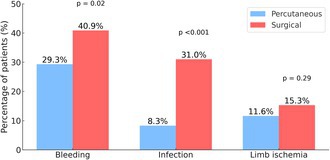

## 1 Introduction

Successful veno‐arterial cannulation is essential for initiating venoarterial extracorporeal membrane oxygenation (V‐A ECMO) in patients with refractory cardiogenic shock, cardiac arrest, or high‐risk cardiac interventions [1, 2, 3, 4]. Femoro‐femoral cannulation, the most common access route, enables rapid V‐A ECMO via percutaneous or surgical techniques [5, 6]. Ultrasound guidance has made percutaneous cannulation the preferred approach in many centers, with studies showing fewer site complications, especially bleeding and infection, than surgical cut‐down [7, 8, 9, 10, 11]. This trend is also evident at our center (Table 1). However, complications still cause morbidity, re‐interventions, and costs regardless of technique [12, 13, 14, 15]. Previous studies focused on selected V‐A ECMO populations [8, 9, 16, 17, 18, 19, 20, 21, 22], and whether their results apply to larger unselected cohorts is still unclear. Confirming this in a real‐world population may improve generalizability and support decisions across heterogeneous groups. We therefore evaluated whether percutaneous or surgical femoro‐femoral V‐A ECMO cannulation was independently associated with site bleeding, infection, and limb ischemia within 90 days in an unselected adult population.

**TABLE 1:** Patient characteristics and procedural details.

	Total (*n* = 384)	Percutaneous group (*n* = 181)	Surgical group (*n* = 203)	*p*
Age (years)	61 (50–69)	61 (49–69)	62 (50–69)	0.82
Male	298 (77.6)	145 (80.1)	153 (75.4)	0.33
BMI (kg/m^2^)	26.9 (24.2–30.1)	26.8 (24.3–30.0)	27.1 (24.2–30.4)	0.46
Comorbidities	307 (79.9)	152 (84.0)	155 (76.4)	0.06
Hypertension	190 (49.5)	86 (47.5)	104 (51.2)	0.47
Hyperlipidemia	131 (34.1)	64 (35.4)	67 (33.0)	0.63
Diabetes mellitus	79 (20.6)	40 (22.1)	39 (19.2)	0.49
Peripheral artery disease	34 (8.9)	12 (6.6)	22 (10.8)	0.15
Coronary artery disease	204 (53.1)	121 (66.9)	83 (40.9)	< 0.001
Congestive heart failure	33 (8.6)	18 (9.9)	15 (7.4)	0.37
Smoking history	104 (27.1)	54 (29.8)	50 (24.6)	0.25
Acute myocardial infarction	164 (42.7)	105 (58.0)	59 (29.1)	< 0.001
Post cardiotomy	158 (41.1)	34 (18.8)	124 (61.1)	< 0.001
ECPR‐cannulation	130 (33.9)	79 (43.6)	51 (25.1)	< 0.001
Periprocedural V‐A ECMO support^a^	20 (5.2)	20 (11.0)	0 (0)	< 0.001
Other	65 (16.9)	33 (18.2)	32 (15.8)	0.52
Platelet inhibitors^b^	109 (28.4)	45 (24.9)	64 (31.5)	0.15
Anticoagulants^b^	133 (34.6)	57 (31.5)	76 (37.4)	0.22
Platelet inhibitors and anticoagulants^b^	48 (12.5)	19 (10.5)	29 (14.3)	0.26
Procedure location				
Operating room	213 (55.5)	49 (27.1)	164 (80.8)	< 0.001
Catheterization laboratory	132 (34.4)	123 (68.8)	9 (4.4)	< 0.001
Intensive care unit	27 (12.0)	8 (4.4)	19 (9.4)	0.06
Emergency department	12 (3.1)	1 (0.6)	11 (5.4)	0.006
Cannulation at OSH and transfer	26 (6.8)	12 (6.6)	14 (6.9)	0.92
Cannulating physician				
Surgeon	275 (71.6)	72 (39.8)	203 (100)	< 0.001
Interventional cardiologist	109 (28.4)	109 (100)	0 (0)	—
Same side arterial/venous cannulation	336 (87.5)	140 (77.3)	196 (96.6)	< 0.001
Arterial cannula, size (Fr)	19 (18–20)	19 (19–19)	19 (18–20)	0.13
Venous cannula, size (Fr)	25 (23–25)	25 (23–25)	25 (23–25)	0.26
Distal perfusion catheter^c^	338 (88.0)	149 (82.3)	189 (93.1)	0.001
Cannulation period				
2007–2013	100 (26.0)	26 (14.4)	74 (36.5)	—
2014–2022	284 (74.0)	155 (85.6)	129 (63.5)	< 0.001

*Note:* Categorical variables are presented as *n* (%) and compared with *χ* ^2^ test. Continuous variables are presented as median (IQR) and compared with Mann–Whitney *U* test. Diagnoses included in the group “Other” (Table S6).

Abbreviations: BMI, body mass index; ECPR, extracorporeal cardiopulmonary resuscitation; OSH, outside hospital.

^a^ V‐A ECMO‐facilitated high‐risk procedures: percutaneous coronary intervention (PCI, *n* = 9), ventricular tachycardia ablation (*n* = 5), transcatheter aortic valve implantation (TAVI, *n* = 4), combined PCI and TAVI (*n* = 2).

^b^ Long‐term treatment before V‐A ECMO.

^c^ At primary cannulation.

## 2 Methods

### 2.1 Study Population

This single‐center study included adults (≥ 18 years) receiving femoro‐femoral V‐A ECMO for refractory cardiogenic shock, cardiac arrest (post‐cardiotomy or nonsurgical), or support during high‐risk cardiac procedures at Karolinska University Hospital (Stockholm, Sweden) between January 2007 and December 2022. Patients were classified by initial cannulation technique; those started percutaneously remained in that group even if converted to cut‐down or surgically decannulated. Inclusion required successful ECMO initiation without other mechanical support. Patients withdrawn for futility were retained, whereas those switched to other cannulation configurations were excluded. Anticoagulation with unfractionated heparin was started if there was no major bleeding, with targets based on indication and paused until controlled if bleeding occurred. Platelet inhibition with P2Y12 inhibitors was added when indicated (e.g., acute coronary syndrome). Acetylsalicylic acid was not combined with P2Y12 inhibitors but could accompany heparin if indicated. Full anticoagulation and antiplatelet protocols are described in File S1. Prophylactic antibiotics were not routinely used post‐cannulation unless administered as part of a routine perioperative regimen in cardiac surgery. Limb perfusion was monitored clinically for signs of ischemia (see Section 2.4) and Doppler assessment of the posterior tibial and dorsalis pedis arteries. Demographics, ECMO characteristics, cannulation‐related complications, and interventions were retrospectively collected and analyzed. This study followed STROBE guidelines for cohort studies [23].

### 2.2 Cannulation Techniques

Thoracic and vascular surgeons performed both surgical and percutaneous cannulations, primarily in the operating room and occasionally in the intensive care unit, while interventional cardiologists performed percutaneous cannulations in the catheterization laboratory. Femoral cannulation involved arterial cannula (15–23 Fr, 23 cm, Maquet, Germany) in the common femoral artery and a venous cannula (21–29 Fr, 55 cm, Maquet, Germany) in the common femoral vein. A distal perfusion cannula (6–8 Fr, Teleflex, NC, USA) was typically placed in the superficial femoral artery to prevent leg ischemia. For percutaneous cannulation, ultrasound‐guided punctures were performed below the inguinal ligament in the ipsilateral (in most cases) or contralateral femoral artery and vein, followed by guidewire insertion using the Seldinger technique, with guidewire positions confirmed by transesophageal echocardiography or fluoroscopy. Stepwise dilation preceded insertion of the arterial return cannula into the descending aorta and the venous drainage cannula near the superior vena cava–right atrium junction. The guidewires were then removed. The distal perfusion cannula was inserted into the superficial femoral artery using the same technique and connected to the arterial cannula [24]. For surgical cannulation, the femoral vessels were exposed through a short vertical groin incision below the inguinal ligament. Purse‐string sutures were applied to the common and superficial femoral arteries and the femoral vein before puncture. Cannulation then proceeded as in the percutaneous technique. After completion, the incision was sutured, and the cannulas were secured. Decannulation was primarily surgical, performed in all surgical and most percutaneously cannulated cases. The arterial cannula was removed first, followed by the distal perfusion cannula. Purse‐string sutures were tied, the femoral artery clamped, and the arteriotomy sutured after inspection for thromboembolic material, with thrombectomy or vascular repair if needed. Finally, the venous cannula was removed, manual compression was applied, and the wound was sutured. Percutaneous V‐A ECMO decannulation, introduced in early 2018 at our center, was an option for percutaneously cannulated patients, using the MANTA device (Teleflex, NC, USA) for arterial cannula removal [25]. The distal perfusion cannula was typically removed with the Angio‐Seal device (Terumo, NJ, USA). The femoral venous cannula was withdrawn, a figure‐of‐eight skin suture tied, and manual pressure applied for 10 min. Final ultrasound or fluoroscopy confirmed adequate distal perfusion and device positioning.

### 2.3 Outcomes

Primary outcomes were cannulation‐site bleeding, site infection, and limb ischemia within 90 days of V‐A ECMO cannulation. Secondary outcomes included interventions related to these complications. The overall incidence of these outcomes was compared between the percutaneous and surgical groups. Both complications and interventions were recorded as present if they occurred at any point within 90 days, regardless of frequency (e.g., multiple bleeding events in one patient were counted as one patient affected by bleeding). The number of patients affected by complications and interventions is shown in Table 2. Hospital and 90‐day survival were assessed for orientation, with 90‐day survival selected over 30‐day survival to capture complications, interventions, and survival in patients requiring extended ECMO support.

**TABLE 2:** Cannulation‐related complications, interventions, and outcomes within 90 days of V‐A ECMO initiation.

	Total (*n* = 384)	Percutaneous group (*n* = 181)	Surgical group (*n* = 203)	*p*
Cannulation‐site bleeding^a^	136 (35.4)	53 (29.3)	83 (40.9)	0.02
Cannula‐site infection^b^	78 (20.3)	15 (8.3)	63 (31.0)	< 0.001
Limb ischemia^c^	52 (13.5)	21 (11.6)	31 (15.3)	0.29
Intervention				
Conversion to surgical cutdown	9 (2.3)	9 (5.0)	n/a	n/a
Cannulation‐site revision^d^	70 (18.2)	22 (12.2)	48 (23.6)	0.004
Vascular surgery^e^	53 (13.8)	20 (11.0)	33 (16.3)	0.14
Thrombectomy	49 (12.8)	16 (8.8)	33 (16.3)	0.03
Cannula relocation	22 (5.7)	10 (5.5)	12 (5.9)	0.87
Fasciotomy	13 (3.4)	4 (2.2)	9 (4.4)	0.23
Amputation	5 (1.3)	2 (1.1)	3 (1.5)	1.00
Overall outcome				
V‐A ECMO duration (days)	5 (2–10)	4 (1–9)	6 (2–12)	< 0.001
Decannulation	250 (65.1)	117 (64.6)	133 (65.5)	0.86
Survival to hospital discharge	180 (46.9)	80 (44.2)	100 (49.3)	0.32
90‐day survival	179 (46.6)	78 (43.1)	101 (49.8)	0.19

*Note:* Categorical variables are presented as *n* (%) and compared with *χ* ^2^ or Fisher's exact test. Variables are not mutually exclusive.

^a^ Major bleeding req. blood transfusion, cannula relocation, surgical cutdown, vascular repair.

^b^ Local infection signs, positive culture of local pathogens, systemic septicemia with local signs.

^c^ Clinical signs, sonographic evidence of limb ischemia, inadequate blood flow.

^d^ Surgical cutdown performed after cannulation or decannulation.

^e^ Patch angioplasty, vascular suturing, desobliteration.

### 2.4 Definitions

Cannulation‐site bleeding was defined as bleeding requiring cannulation strategy modification (cannula relocation, conversion to surgical cut‐down), site revision, vascular repair (patch angioplasty, suturing, stenting), or blood transfusion when clinically indicated. Minor bleeding, oozing, and local hematoma were not recorded. Cannulation‐site infection was defined by local infection signs (inflammation, purulent drainage, wound dehiscence) with positive pathogen cultures or systemic septicemia; limb ischemia was defined as clinical signs of ischemia (pallor, poikilothermia, pulselessness) or sonographic evidence of insufficient or absent distal perfusion. Full definitions are provided in Table S1.

### 2.5 Statistical Analyses

Continuous variables were tested for normality using the Kolmogorov–Smirnov test, confirmed as nonnormally distributed, and presented as medians with interquartile ranges (IQR). Categorical variables were presented as numbers and percentages. Comparisons between the percutaneous and surgical groups used the Mann–Whitney *U* test for continuous variables and the *χ* ^2^ or Fisher's exact test (if sample size < 5) for categorical variables. Logistic regression assessed whether cannulation technique was independently associated with the three primary outcomes: site bleeding, infection, and limb ischemia within 90 days after cannulation. Separate models were generated for each outcome to assess associations with cannulation technique and pre‐specified variables (Tables S2–S4). Variables with *p* < 0.05 in univariable analysis were tested for multicollinearity using variance inflation factors (VIFs) before inclusion in multivariable models, except when event timing relative to the outcome was uncertain. To ensure only variables that preceded the outcomes were included in the regression analyses, site infection, site revision, and limb ischemia were excluded from the bleeding model, as it could not be confirmed whether these variables occurred before or as a result of bleeding. Similarly, site revision was excluded from the infection model, and bleeding, infection, and site revision were excluded from the ischemia model, as it could not be verified that these variables always occurred before the outcomes. No variables in the regression analyses had missing data. Model fit was assessed using the Hosmer–Lemeshow test. Logistic regression results are presented as odds ratios (ORs) with 95% confidence intervals (CIs), reflecting the strength of association between the independent variables and the outcomes, rather than predicting individual patient risk. All tests were two‐sided, with significance set at *p* < 0.05. Analyses were conducted in SPSS (version 28, IBM, New York, NY).

## 3 Results

### 3.1 Patients

A total of 384 patients supported with femoro‐femoral V‐A ECMO were enrolled. No patients were lost to follow‐up. Demographics and ECMO details are summarized and compared between percutaneous and surgical groups (Table 1).

Percutaneous cannulation was performed in 181 (47.1%) patients, and 203 (52.9%) had surgical cannulation. Median age, gender, body mass index (BMI), and most comorbidities did not differ significantly, except for coronary artery disease, which was more common in the percutaneous group (66.9% vs. 40.9%, *p* < 0.001). Percutaneous patients more often presented with acute myocardial infarction (58.0% vs. 29.1%, *p* < 0.001), and refractory cardiac arrest (ECPR‐cannulation) (43.6% vs. 25.1%, *p* < 0.001). In contrast, the surgical group had more prior cardiac surgery (61.1% vs. 18.8%, *p* < 0.001), received distal perfusion catheterization at primary cannulation more often (93.1% vs. 82.3%, *p* = 0.001), and all were cannulated by surgeons, mainly in the operating room (Table 1). Arterial cannula size did not differ between percutaneous and surgical cannulations (median 19 Fr [IQR 19–19] vs. 19 Fr [IQR 18–20]; *p* = 0.13; Table 1).

### 3.2 Cannulation‐Related Complications and Interventions

**FIGURE 1:**
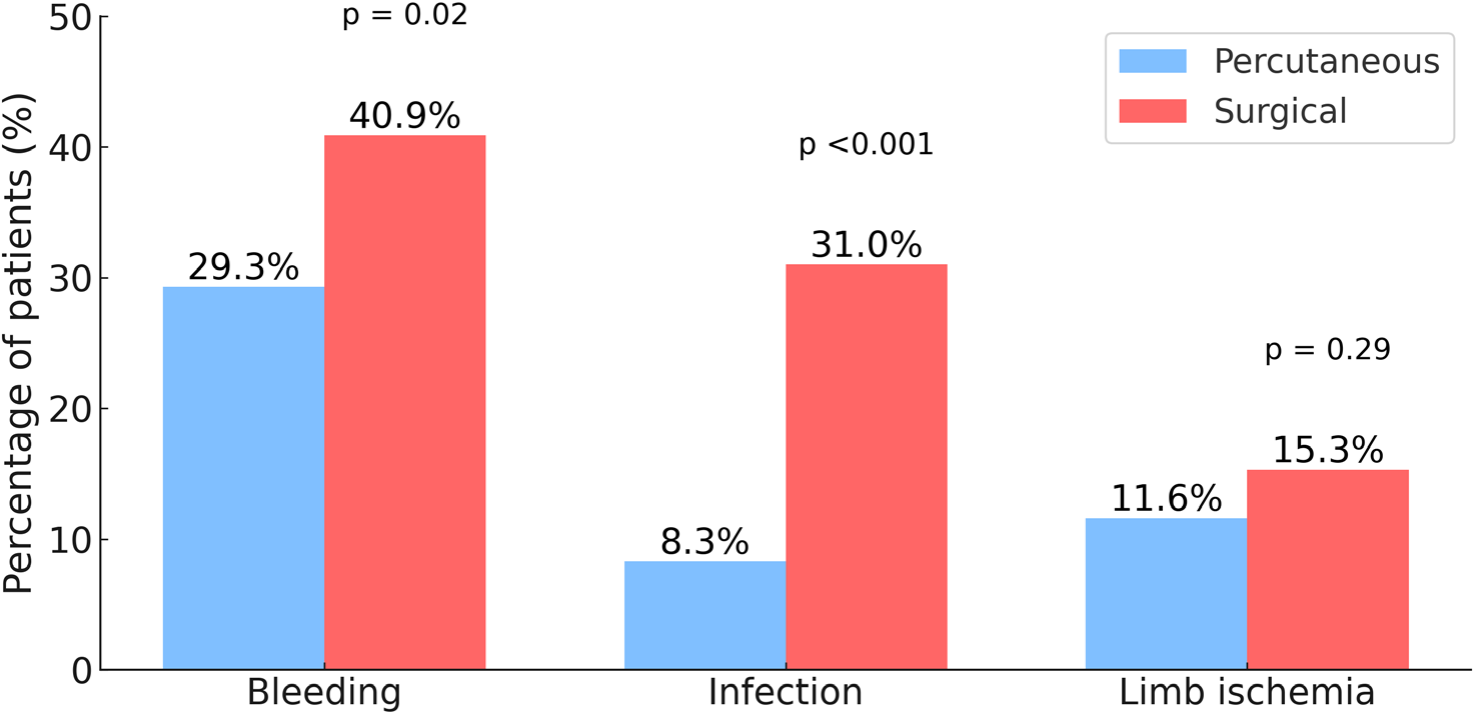
Percentages of patients with cannulation‐site bleeding, infection, and limb ischemia by cannulation technique within 90 days of cannulation. [Color figure can be viewed at wileyonlinelibrary.com]

Overall, the most frequent complication was site bleeding (35.4% of the study population), followed by site infection (20.3%) and limb ischemia (13.5%). Fewer patients in the percutaneous group experienced bleeding (29.3% vs. 40.9%, *p* = 0.02) and infection (8.3% vs. 31.0%, *p* < 0.001), while the number with limb ischemia was similar (11.6% vs. 15.3%, *p* = 0.29) (Figure 1).

During cannulation, 5% (*n* = 9) of percutaneous patients required conversion to surgical cutdown. Nearly twice as many surgical patients underwent site revision and thrombectomy (23.6% vs. 12.2%, *p* = 0.004; and 16.3% vs. 8.8%, *p* = 0.03), whereas vascular surgery, cannula relocation, lower‐extremity fasciotomy, and below‐knee amputation did not differ between groups (Table 2).

### 3.3 Factors Associated With Site Bleeding, Infection, and Limb Ischemia

#### 3.3.1 Logistic Regression Models

Three multivariable logistic regression models were used to assess variables independently associated with site bleeding, infection, and limb ischemia within 90 days of V‐A ECMO cannulation (Table 3). All variables included in the models had VIFs < 1.5, confirming the absence of multicollinearity.

**TABLE 3:** Regression analyses of factors associated with cannulation‐site complications.

	Univariable logistic regression		Multivariable logistic regression	
	OR (95% CI)	*p*	OR (95% CI)	*p*
Cannulation‐site bleeding				
Arterial complication at cannulation^a^	6.17 (2.58–14.77)	< 0.001	5.97 (2.42–14.75)	< 0.001
Surgical cannulation	2.81 (1.73–4.55)	< 0.001	2.39 (1.43–3.98)	< 0.001
V‐A ECMO duration (days)	1.07 (1.04–1.10)	< 0.001	1.07 (1.03–1.10)	< 0.001
Cannulation‐site infection				
Surgical cannulation	6.02 (3.14–11.53)	< 0.001	5.47 (2.47–12.12)	< 0.001
Cannulation‐site bleeding^b^	3.37 (1.93–5.90)	< 0.001	2.81 (1.42–5.55)	0.003
BMI (kg/m^2^)	1.11 (1.04–1.17)	< 0.001	1.13 (1.05–1.21)	0.002
V‐A ECMO duration (days)	1.13 (1.08–1.18)	< 0.001	1.10 (1.05–1.16)	< 0.001
ECPR‐cannulation	0.38 (0.18–0.80)	0.01	0.57 (0.24–1.38)	0.22
Post cardiotomy	1.89 (1.10–3.25)	0.02	0.76 (0.37–1.57)	0.46
Limb ischemia				
Arterial complication at cannulation^a^	9.77 (4.11–23.24)	< 0.001	13.97 (5.14–37.96)	< 0.001
Absence of distal perfusion catheter	2.15 (1.02–4.56)	0.046	3.59 (1.49–8.67)	0.005
Arterial cannula size (Fr)	1.31 (1.02–1.68)	0.03	1.55 (1.18–2.04)	0.002
V‐A ECMO duration (days)	1.05 (1.02–1.09)	0.002	1.07 (1.03–1.11)	< 0.001

Abbreviations: BMI, body mass index; CI, confidence interval; ECPR extracorporeal cardiopulmonary resuscitation; OR, odds ratio.

^a^ Vessel laceration, multiple vessel punctures, femoral dissection.

^b^ Major bleeding req. blood transfusion, cannula relocation, surgical cutdown, vascular repair. All bleeding started before site infection.

#### 3.3.2 Bleeding

The model was statistically significant, *χ* ^2^ (3, *n* = 384) = 53.061; *p* < 0.001, distinguishing between patients with and without site bleeding. The Hosmer–Lemeshow test (*χ* ^2^ = 11.791, *p* = 0.16) confirmed good model fit. The model explained 12.9% (Cox & Snell *R* ^2^) to 18.5% (Nagelkerke *R* ^2^) of the variance and correctly classified 73.4% of cases. Three variables remained significant in multivariable logistic regression: arterial complications at cannulation (OR 5.97; 95% CI 2.42–14.75; *p* < 0.001), surgical cannulation (OR 2.39; 95% CI 1.43–3.98; *p* < 0.001), and ECMO duration per day increase (OR 1.07; 95% CI 1.03–1.10; *p* < 0.001). Surgical cannulation, adjusted for the other two variables in the model, was associated with a 2.4‐fold increased risk of site bleeding.

#### 3.3.3 Infection

The model was statistically significant, *χ* ^2^ (8, *n* = 384) = 84.624; *p* < 0.001, distinguishing between patients with and without site infection. The Hosmer–Lemeshow test (*χ* ^2^ = 6.591, *p* = 0.37) confirmed good model fit. The model explained 29.1% (Cox & Snell *R* ^2^) to 41.2% (Nagelkerke *R* ^2^) of the variance in infection and correctly classified 76.6% of cases. In multivariable logistic regression, four variables remained significant: surgical cannulation (OR 5.47; 95% CI 2.47–12.12; *p* < 0.001), site bleeding (OR 2.81; 95% CI 1.42–5.55; *p* = 0.003), BMI per unit increase (OR 1.13; 95% CI 1.05–1.21; *p* = 0.002), and ECMO duration per day (OR 1.10; 95% CI 1.05–1.16; *p* < 0.001). Surgical cannulation, adjusted for the other variables in the model, was associated with a 5.5‐fold higher risk of site infection.

#### 3.3.4 Limb Ischemia

The model was statistically significant, *χ* ^2^ (6, *n* = 384) = 47.816; *p* < 0.001, distinguishing between patients with and without limb ischemia. The Hosmer–Lemeshow test (*χ* ^2^ = 6.358, *p* = 0.61) confirmed good model fit. The model explained 12.2% (Cox & Snell *R* ^2^) to 24.5% (Nagelkerke *R* ^2^) of the variance in limb ischemia and correctly classified 90.0% of cases. In multivariable logistic regression, four independent variables significantly contributed to the model, but cannulation technique did not. Arterial complication at cannulation was the strongest risk factor for limb ischemia (OR 13.97; 95% CI 5.14–37.96; *p* < 0.001), followed by absence of distal perfusion catheter (OR 3.59; 95% CI 1.49–8.67; *p* = 0.005), arterial cannula size per French increase (OR 1.55; 95% CI 1.18–2.04; *p* = 0.002), and ECMO duration per day (OR 1.07; 95% CI 1.03–1.11; *p* < 0.001). While cannulation technique was not an independent risk factor for limb ischemia, modifiable factors such as absence of distal perfusion catheterization at primary cannulation and larger arterial cannula size, were associated with a 3.6‐fold and 1.6‐fold increased risk of limb ischemia, respectively, adjusted for the other variables in the model.

### 3.4 Overall Outcome

The median V‐A ECMO duration was shorter in the percutaneous group than in the surgical group (4 days, IQR 1–9 vs. 6 days, IQR 2–12; *p* < 0.001). Twenty patients (5.2% of the total cohort) were supported > 21 days, with a maximum of 52 days in the percutaneous group and 38 in the surgical group. Decannulation rates and survival outcomes, including survival to discharge and 90‐day survival, did not differ significantly between groups (Table 2). No deaths were attributed to the cannulation or decannulation procedures. Although the primary focus of this study was cannulation‐related complications, additional comparisons between percutaneous and surgical decannulation techniques are provided in Table S5 for orientation.

## 4 Discussion

To our knowledge, this is one of the largest studies to assess cannulation‐related complications in femoro‐femoral V‐A ECMO and the first to include an unselected cohort. The main finding was that percutaneous cannulation was associated with a significantly lower risk of site bleeding and infection compared with surgical cannulation, whereas no significant association was found between limb ischemia and cannulation technique. These findings align with earlier reports showing a higher proportion of patients with bleeding and infection after surgical cannulation [8, 9]. Among the modifiable factors that can be considered at cannulation beyond the choice of technique, two key variables were identified: absence of distal perfusion catheterization and increasing arterial cannula size (with cannula size similar across techniques), both associated with a higher risk of limb ischemia. These findings agree with previous reports highlighting the protective role of distal perfusion catheters and the association between larger arterial cannulas and limb ischemia, and extend these results to an unselected cohort in which these procedural variables were evaluated alongside technique [19, 26, 27]. This shows that these two variables should be considered together with cannulation technique to reduce ischemia risk, as they may play a more direct role than the technique itself. In addition to surgical cannulation, BMI, ECMO duration, and arterial complications were variables associated with a higher proportion of patients with bleeding and infection, though they are largely nonmodifiable at cannulation.

Given the differences in definitions, patient selection, reporting of missing data, and exclusion criteria, direct comparisons with previous studies are challenging [8, 9, 14, 18, 19, 27, 28, 29]. Several studies examine either percutaneous or surgical techniques without directly comparing complications, or include ECMO modes beyond femoro‐femoral access [14, 17, 19, 20, 21]. Others exclude patients who died during ECMO, received ECPR cannulation, or used propensity matching, which may omit a significant portion of patients and limit comparability [8, 16, 22].

Comparison of bleeding severity is difficult because of heterogeneous cohorts and varying definitions, including hemoglobin drop criteria [30]. We did not include hemoglobin drop in our definition, as thresholds vary across subgroups (e.g., postoperative, acute coronary syndrome, etc.). For instance, a baseline hemoglobin level of 7 g/dL may prompt earlier transfusion and intervention compared with a baseline of 12 g/dL, making a fixed drop (e.g., > 3 g/dL as in the Bleeding Academic Research Consortium [BARC] criteria 3A‐B) potentially misleading and risking underreporting. Furthermore, BARC criteria were designed for antithrombotic therapy trials, mainly for evaluating coronary stents, not for unselected patients with large‐bore cannulas and heparin ± platelet inhibitors [30].

Instead, we defined cannulation‐site bleeding as major bleeding requiring intervention, independent of hemoglobin drop. Intervention was judged to be a more objective threshold for defining bleeding severity, as an intervention may occur before a specific hemoglobin drop is reached. This approach ensures that all patients requiring resource‐intensive interventions for bleeding are included, which may explain the higher proportion of patients with bleeding reported in our study compared with other reports [15].

Compared with our cohort, Danial et al. used propensity score matching to balance baseline characteristics, reducing confounding and simulating a more randomized design [8]. Although matching clarifies comparisons, many patients were excluded (45% surgical and 19% percutaneous in that study), which can bias results if excluded patients differ in characteristics or outcomes. Residual confounding remains, as propensity matching only addresses observed variables, leaving unmeasured confounders unaccounted for. Our nonmatched design included all eligible patients, preserving real‐world applicability and avoiding bias from exclusions.

Regarding patients who did not survive to decannulation, we chose not to exclude them, unlike other studies [16, 31]. Excluding them would underreport complications, as these patients still required interventions. Including all patients allowed assessment of complications both early and after ECMO, even in those who did not survive to decannulation. Table S7 illustrates potential underreporting if nondecannulated patients were excluded.

Although survival differences between groups were not found, survival‐related factors were outside the scope of this study, which focused on the associations between cannulation technique and site bleeding, infection, and limb ischemia. The population was heterogeneous, from patients requiring periprocedural support to those with refractory cardiac arrest undergoing ECPR‐cannulation. Consequently, survival rates may differ across subgroups, but we did not analyze this beyond technique. Survival data should therefore not be interpreted only in relation to technique. The observed rates of successful decannulation (65.1%), in‐hospital mortality (53.1%), and discharge alive (46.9%) match those reported in the pooled analysis by Jia et al., which included 42 studies and 6164 patients receiving peripheral venoarterial ECMO (60.6%, 54.1%, and 44.6%, respectively) [15].

Tracking complications over 90 days captured early and late events, including in patients with prolonged ECMO, extending beyond the commonly reported 30‐day follow‐up [8, 9]. This provides a more comprehensive view of cannulation‐related complications, particularly site infections after decannulation (Table S5).

Even though our findings support the growing use of percutaneous access, surgical backup remains essential for managing cannulation‐site complications, particularly when percutaneous cannulation is used.

## 5 Limitations

The retrospective design of this study introduces inherent biases and limitations. First, it relied on single‐center definitions for site‐specific complications and interventional protocols, with all involved interventionists and surgeons having extensive experience inserting large‐bore cannulas with both percutaneous and surgical techniques, which may limit generalizability. Second, the increasing number of ECMO cases over the 16‐year study period, including the rising use of percutaneous cannulation, may have introduced temporal variability, which was not analyzed. Third, the absence of routine follow‐up imaging (e.g., ultrasound or computed tomography) may have led to underreporting of complications such as subclinical stenosis, arteriovenous fistula, and pseudoaneurysm. Fourth, complications occurring beyond 90 days were not captured, potentially leading to further underreporting of late complications such as persistent limb sensory‐motor deficit. Fifth, the impact of vasopressor support on limb ischemia could not be assessed. Sixth, the role of pre‐existing peripheral artery disease in limb ischemia may have been underestimated due to the lack of routine screening. Finally, while logistic regression adjusted for known confounders, residual confounding may still influence the observed associations.

## 6 Conclusion

This large retrospective study found that percutaneous cannulation was associated with more than a two‐fold lower risk of site bleeding and close to six‐fold lower risk of infection compared with the surgical technique for femoro‐femoral V‐A ECMO cannulation. Although cannulation technique itself was not associated with limb ischemia, the absence of distal perfusion catheterization at primary cannulation and larger arterial cannula size were, underscoring the importance of considering these two modifiable factors in addition to the choice of cannulation technique. These findings, derived from an unselected patient population, may facilitate decision‐making in the everyday clinical management of patients considered for bi‐femoral V‐A ECMO.

## Author Contributions

Concept/design, data collection, data analysis/interpretation, and drafting of the manuscript: Magnus Dalén, Axel Dimberg, Thomas Fux. Critical revision for important intellectual content: Magnus Dalén, Axel Dimberg, Anders Franco‐Cereceda, Thomas Fux. All authors approved the final version of the manuscript.

## Ethics Statement

This study was approved by the Regional Ethics Review Board in Stockholm (registration no. 2021‐04822, 2025‐04356‐02). Informed consent was waived for this retrospective study, as it did not modify existing diagnostic or therapeutic strategies.

## Consent

The authors have nothing to report.

## Conflicts of Interest

The authors declare no conflicts of interest.

## Supporting information


**File S1:** Anticoagulation and platelet inhibition.

**Table S1:** Variable definitions.

**Table S2:** Regression analyses of factors associated with cannulation‐site bleeding.
**Table S3:** Regression analyses of factors associated with cannulation‐site infection.
**Table S4:** Regression analyses of factors associated with limb ischemia.

**Table S5:** Cannulation‐related complications and interventions during decannulation and post‐decannulation periods.

**Table S6:** Patients with diagnoses classified as “Other.”

**Table S7:** Nondecannulated patients with complications and interventions.

## Data Availability

The data that support the findings of this study are available on request from the corresponding author. The data are not publicly available due to privacy or ethical restrictions.
