# Supplementary material for: Percutaneous Versus Surgical Cannulation for Femoro‐Femoral Venoarterial Extracorporeal Membrane Oxygenation: A Retrospective Cohort Study on Cannulation‐Related Complications
Source: Artif Organs. 2025 Nov 21;50(3):440–8. doi: 10.1111/aor.70061 (PMC13090744; doi:10.1111/aor.70061)
Supplement: Supplementary file 1 — File S1: Anticoagulation and platelet inhibition. [file AOR-50-440-s006.docx]

**FILE S1 Anticoagulation and platelet inhibition**

**Anticoagulation**

- Bolus dose of unfractionated heparin (UFH): A bolus of 75–100 IU/kg UFH is routinely administered intravenously at cannulation, typically after guidewire placement. Continuous intravenous UFH infusion is generally delayed for at least 24–36 hours, unless there is evidence of massive pulmonary embolism, thrombotic material in the circuit, or intracardiac thrombi. The delay may be extended for several days depending on ongoing bleeding or high bleeding risk, such as post-surgery, following cardiopulmonary resuscitation, or during concurrent P2Y12 inhibitor therapy (e.g., cangrelor, prasugrel, ticagrelor, clopidogrel).
- **Continuous infusion of UFH: When no significant bleeding is present and rotational thromboelastometry (ROTEM) findings are near-normal, a starting dose of 5–10 IU/kg/hour is initiated. The infusion rate is adjusted to maintain activated partial thromboplastin time (aPTT) or anti-factor Xa (anti-FXa) levels within target ranges, depending on clinical indication (see below). We do not routinely tailor the UFH dose based on activated clotting time (ACT) during ECMO, except before or during weaning, or if cannulas must be clamped to stop flow through the ECMO circuit.**

**The aPTT target intervals**

The aPTT is monitored using the Actin FS activated partial thromboplastin time reagent (normal reference range: 20–30 seconds). Target aPTT intervals are as follows:

- **Routine thromboprophylaxis** (for surgical/non-surgical patients or those on P2Y12 inhibitors): 40–60 seconds
- **Therapeutic anticoagulation** (e.g., for prosthetic heart valves, left ventricular thrombus, spontaneous echo contrast in left heart chambers/aortic root, atrial fibrillation): 50–80 seconds

The lower end of the range is preferred in patients at high risk of bleeding (e.g., recent surgery or impaired coagulation), while the upper end is used in patients at high thromboembolic risk or requiring active bleeding control. For high-risk conditions (e.g., massive pulmonary embolism, ECMO circuit thrombosis, antiphospholipid syndrome, severe sepsis, platelets >300 × 10⁹/L with hyperactive platelet function, as shown by ROTEM), a target aPTT of **80–100 seconds** may be used.

**Anti-FXa target intervals**

The Anti-FXa monitoring serves as an alternative or adjunct to aPTT, with routine target ranges:

- **Thromboprophylaxis: 0.2–0.4 IU/mL**
- **Therapeutic anticoagulation (**for high-risk conditions as described): **0.5–0.7 IU/mL**

Anti-FXa levels are monitored as needed or every 12 hours during transition from aPTT monitoring.

**Heparin-induced thrombocytopenia (HIT Type II)**

In cases of HIT, direct thrombin inhibitors are used. Argatroban is the first-line agent, and bivalirudin is second-line, depending on renal and hepatic function. Therapy is monitored using aPTT, ROTEM, or ACT, applying the same target ranges as during heparin therapy. Since 2022, argatroban is primarily monitored using plasma argatroban levels (enzymatic anti-factor IIa assay), with a target range of 0.5–1.5 mg/L.

**Additional routine anticoagulation parameters**

- **Antithrombin levels: 0.8–1.2 IU/mL**
- **Fibrinogen: >200 mg/dL**
- **Hemoglobin: >9.0 g/dL**
- **Platelet count: >80 × 10⁹/L** (increased to >100 × 10⁹/L for transfusion-requiring bleeding or patients on P2Y12 inhibitors)

Transfusions are administered restrictively, with individualized thresholds for hemoglobin and platelet counts, particularly in patients being considered for transplantation.

**Platelet Inhibition**

Antiplatelet therapy during ECMO is tailored to the patient’s bleeding risk and clinical indications, such as P2Y12 inhibitors (cangrelor, prasugrel, ticagrelor, clopidogrel) in acute coronary syndrome, or post-percutaneous coronary intervention (PCI) with stenting or balloon angioplasty.

Acetylsalicylic acid is not used concurrently with P2Y12 inhibitors during ECMO but is used as the sole platelet inhibitor if no PCI was performed, stable coronary artery disease, or prior to or after coronary artery bypass grafting. After weaning from ECMO support, dual antiplatelet therapy is introduced as indicated.
