# Supplementary material for: Percutaneous Versus Surgical Cannulation for Femoro‐Femoral Venoarterial Extracorporeal Membrane Oxygenation: A Retrospective Cohort Study on Cannulation‐Related Complications
Source: Artif Organs. 2025 Nov 21;50(3):440–8. doi: 10.1111/aor.70061 (PMC13090744; doi:10.1111/aor.70061)
Supplement: Supplementary file 2 — Table S1: Variable definitions. [file AOR-50-440-s002.docx]

**TABLE S1** Variable definitions

|  | Definitions |
| --- | --- |
| Arterial vascular complication | Vessel laceration, obstruction, dissection, or multiple vessel punctures related to cannulation. |
| Cannulation-site bleeding | Major bleeding requiring cannulation strategy modification (cannula relocation, conversion to surgical cutdown), site revision after completed cannulation or decannulation, vascular repair (patch angioplasty, vascular suturing, stenting), blood transfusion. Minor bleeding, oozing, and local hematoma were not recorded. |
| Cannulation-site infection | Local signs of infection (inflammation, purulent drainage, wound dehiscence) with positive cultures of local pathogens, or systemic septicemia with local infection signs. |
| Cannulation-site revision | Surgical cutdown after completed cannulation or decannulation to address complication (site bleeding, hematoma, infection, limb ischemia, thrombosis, pseudoaneurysm, site necrosis, cannula dislocation), which may involve vascular surgery (patch angioplasty, vascular suturing, desobliteration, resection of pseudoaneurysm) as part of the revision process. |
| Cannulation strategy modification | Cannula relocation, conversion to surgical cutdown at percutaneous cannulation or decannulation. |
| Coronary artery disease | AMI, unstable angina pectoris at admission, verified by coronary angiography or CCTA, prior AMI, or evidence of coronary artery disease based on previous coronary angiography, CCTA, or a history of PCI or CABG prior to index hospitalization. |
| ECPR-cannulation | Cannulation performed during ongoing cardiopulmonary resuscitation. |
| Limb ischemia | Clinical signs of ischemia in the arterially cannulated leg (pallor, poikilothermia, pulselessness), and sonographic evidence of inadequate or absent distal perfusion. |
| Limb sensory-motor deficit | Peripheral nerve injury with sensory-motor deficit in lower limb originating from cannulation groin. |
| Periprocedural V-A ECMO support | V-A ECMO-facilitated high-risk interventional procedures: PCI, ventricular tachycardia ablation, TAVI, combined PCI and TAVI. |
| Post cardiotomy | Cardiac surgery prior to V-A ECMO during the current (index) hospitalization. |
| Post-decannulation | The period after ECMO decannulation, with surviving patients followed until 90 days after cannulation. |
| Venous vascular complication | Vessel perforation, laceration, related to cannulation. |

Abbreviations: AMI, acute myocardial infarction; CABG, coronary artery bypass grafting; CCTA, coronary computed tomography angiography; PCI, percutaneous coronary intervention; TAVI, transcatheter aortic valve implantation.
