# Supplementary material for: Percutaneous Versus Surgical Cannulation for Femoro‐Femoral Venoarterial Extracorporeal Membrane Oxygenation: A Retrospective Cohort Study on Cannulation‐Related Complications
Source: Artif Organs. 2025 Nov 21;50(3):440–8. doi: 10.1111/aor.70061 (PMC13090744; doi:10.1111/aor.70061)
Supplement: Supplementary file 3 — Table S2: Regression analyses of factors associated with cannulation‐site bleeding. Table S3: Regression analyses of factors associated with cannulation‐site infection. Table S4: Regression analyses of factors associated with limb ischemia. [file AOR-50-440-s004.docx]

**TABLE S2** Regression analyses of factors associated with cannulation-site bleeding

|  | **Univariable logistic**  **regression** | | **Multivariable logistic regression** | |
| --- | --- | --- | --- | --- |
|  | **OR (95% CI)** | ***p*** | **OR (95% CI)** | ***p*** |
| Age (years) | 0.99 (0.97-1.00) | 0.07 |  |  |
| Male | 0.83 (0.49-1.40) | 0.48 |  |  |
| BMI (kg/m^2^) | 0.98 (0.94-1.02) | 0.36 |  |  |
| Hypertension | 0.91 (0.58-1.41) | 0.66 |  |  |
| Hyperlipidemia | 1.31 (0.83-2.08) | 0.25 |  |  |
| Diabetes mellitus | 0.97 (0.56-1.68) | 0.91 |  |  |
| Peripheral artery disease | 0.76 (0.33-1.73) | 0.51 |  |  |
| Coronary artery disease | 0.74 (0.47-1.15) | 0.18 |  |  |
| Congestive heart failure | 0.94 (0.41-2.01) | 0.88 |  |  |
| Smoking history | 0.59 (0.59-1.60) | 0.89 |  |  |
| Acute myocardial infarction | 0.90 (0.62-1.52) | 0.94 |  |  |
| Post cardiotomy | 0.77 (0.49-1.22) | 0.27 |  |  |
| ECPR-cannulation | 1.01 (0.63-1.61) | 0.98 |  |  |
| Platelet inhibitors | 1.14 (0.70-1.85) | 0.61 |  |  |
| Anticoagulants | 1.13 (0.17-1.80) | 0.59 |  |  |
| Platelet inhibitors and anticoagulants | 1.05 (0.54-2.03) | 0.90 |  |  |
| Arterial cannula, size (Fr) | 0.92 (0.71-1.18) | 0.51 |  |  |
| Venous cannula, size (Fr) | 1.10 (0.94-1.29) | 0.25 |  |  |
| Distal perfusion catheter, size (Fr) | 0.93 (0.72-1.21) | 0.60 |  |  |
| Distal perfusion catheter | 1.29 (0.68-2.47) | 0.44 |  |  |
| Surgical cannulation | 2.81 (1.73-4.55) | <0.001 | 2.39 (1.43-3.98) | <0.001 |
| Same side arterial/venous cannulation | 0.96 (0.49-1.86) | 0.90 |  |  |
| Arterial complication at cannulation | 6.17 (2.58-14.77) | <0.001 | 5.97 (2.42-14.75) | <0.001 |
| Venous complication at cannulation | 2.57 (0.51-12.91) | 0.25 |  |  |
| V-A ECMO duration (days) | 1.07 (1.04-1.10) | <0.001 | 1.07 (1.03-1.10) | <0.001 |

Abbreviations: BMI, body mass index; CI, confidence interval; ECPR, extracorporeal cardiopulmonary resuscitation; OR, odds ratio. Full definitions are provided in Supporting Information Table S1.

**TABLE S3** Regression analyses of factors associated with cannulation-site infection

|  | **Univariable logistic**  **regression** | | **Multivariable logistic regression** | |
| --- | --- | --- | --- | --- |
|  | **OR (95% CI)** | ***p*** | **OR (95% CI)** | ***p*** |
| Age (years) | 1.00 (0.98-1.02) | 0.75 |  |  |
| Male | 0.96 (0.50-1.83) | 0.89 |  |  |
| BMI (kg/m^2^) | 1.11 (1.04-1.17) | <0.001 | 1.13 (1.05-1.21) | 0.002 |
| Hypertension | 0.90 (0.53-1.54) | 0.71 |  |  |
| Hyperlipidemia | 0.95 (0.54-1.66) | 0.86 |  |  |
| Diabetes mellitus | 1.21 (0.63-2.33) | 0.57 |  |  |
| Peripheral artery disease | 0.94 (0.32-2.75) | 0.90 |  |  |
| Coronary artery disease | 0.69 (0.41-1.19) | 0.18 |  |  |
| Congestive heart failure | 0.48 (0.18-1.32) | 0.15 |  |  |
| Smoking history | 1.50 (0.84-2.68) | 0.17 |  |  |
| Acute myocardial infarction | 0.68 (0.39-1.18) | 0.17 |  |  |
| Post cardiotomy | 1.89 (1.10-3.25) | 0.02 | 0.76 (0.37-1.57) | 0.46 |
| ECPR-cannulation | 0.38 (0.18-0.80) | 0.01 | 0.57 (0.24-1.38) | 0.22 |
| Platelet inhibitors | 1.22 (0.69-2.18) | 0.50 |  |  |
| Anticoagulants | 1.06 (0.61-1.84) | 0.85 |  |  |
| Platelet inhibitors and anticoagulants | 0.72 (0.32-1.61) | 0.42 |  |  |
| Arterial cannula, size (Fr) | 1.01 (0.83-1.22) | 0.95 |  |  |
| Venous cannula, size (Fr) | 1.15 (0.95-1.38) | 0.15 |  |  |
| Distal perfusion catheter, size (Fr) | 1.00 (0.73-1.37) | 0.99 |  |  |
| Distal perfusion catheter | 0.86 (0.44-1.69) | 0.67 |  |  |
| Surgical cannulation | 6.02 (3.14-11.53) | <0.001 | 5.47 (2.47-12.12) | <0.001 |
| Same side arterial/venous cannulation | 2.51 (0.83-7.56) | 0.13 |  |  |
| Arterial complication at cannulation | 2.01 (0.65-6.20) | 0.22 |  |  |
| Venous complication at cannulation | 1.13 (0.10-12.65) | 0.92 |  |  |
| Cannulation-site bleeding | 3.37 (1.93-5.90) | <0.001 | 2.81 (1.42-5.55) | 0.003 |
| Limb ischemia | 1.41 (0.64-3.11) | 0.40 |  |  |
| V-A ECMO duration (days) | 1.13 (1.08-1.18) | <0.001 | 1.10 (1.05-1.16) | <0.001 |

Abbreviations: BMI, body mass index; CI, confidence interval; ECPR, extracorporeal cardiopulmonary resuscitation; OR, odds ratio. Full definitions are provided in Supporting Information Table S1.

**TABLE S4** Regression analyses of factors associated with limb ischemia

|  | **Univariable logistic**  **regression** | | **Multivariable logistic regression** | |
| --- | --- | --- | --- | --- |
|  | **OR (95% CI)** | ***p*** | **OR (95% CI)** | ***p*** |
| Age (years) | 0.99 (0.97-1.01) | 0.26 |  |  |
| Male | 2.37 (0.90-6.22) | 0.08 |  |  |
| BMI (kg/m^2^) | 1.04 (0.98-1.10) | 0.18 |  |  |
| Hypertension | 0.97 (0.52-1.83) | 0.93 |  |  |
| Hyperlipidemia | 1.45 (0.76-2.77) | 0.26 |  |  |
| Diabetes mellitus | 1.58 (0.77-3.25) | 0.21 |  |  |
| Peripheral artery disease | 2.26 (0.92-5.56) | 0.08 |  |  |
| Coronary artery disease | 1.26 (0.66-2.39) | 0.49 |  |  |
| Congestive heart failure | 0.78 (0.23-2.66) | 0.69 |  |  |
| Smoking history | 1.52 (0.78-2.97) | 0.22 |  |  |
| Acute myocardial infarction | 0.96 (0.51-1.83) | 0.91 |  |  |
| Post cardiotomy | 1.58 (0.84-2.98) | 0.16 |  |  |
| ECPR-cannulation | 1.05 (0.54-2.05) | 0.88 |  |  |
| Platelet inhibitors | 0.97 (0.48-1.97) | 0.94 |  |  |
| Anticoagulants | 0.80 (0.40-1.59) | 0.52 |  |  |
| Platelet inhibitors and anticoagulants | 0.69 (0.24-2.03) | 0.50 |  |  |
| Arterial cannula, size (Fr) | 1.31 (1.02-1.68) | 0.03 | 1.55 (1.18-2.04) | 0.002 |
| Venous cannula, size (Fr) | 1.15 (0.92-1.44) | 0.22 |  |  |
| Distal perfusion catheter, size (Fr) | 0.90 (0.61-1.32) | 0.90 |  |  |
| Absence of distal perfusion catheter | 2.15 (1.02-4.56) | 0.046 | 3.59 (1.49-8.67) | 0.005 |
| Surgical cannulation | 1.59 (0.82-3.01) | 0.17 |  |  |
| Same side arterial/venous cannulation | 0.70 (0.29-1.68) | 0.43 |  |  |
| Arterial complication at cannulation | 9.77 (4.11-23.24) | <0.001 | 13.97 (5.14-37.96) | <0.001 |
| Venous complication at cannulation | 4.11 (0.73-23.14) | 0.11 |  |  |
| V-A ECMO duration (days) | 1.05 (1.02-1.09) | 0.002 | 1.07 (1.03-1.11) | <0.001 |

Abbreviations: BMI, body mass index; CI, confidence interval; ECPR, extracorporeal cardiopulmonary resuscitation; OR, odds ratio. Full definitions are provided in Supporting Information Table S1.
