# Supplementary material for: Percutaneous Versus Surgical Cannulation for Femoro‐Femoral Venoarterial Extracorporeal Membrane Oxygenation: A Retrospective Cohort Study on Cannulation‐Related Complications
Source: Artif Organs. 2025 Nov 21;50(3):440–8. doi: 10.1111/aor.70061 (PMC13090744; doi:10.1111/aor.70061)
Supplement: Supplementary file 4 — Table S5: Cannulation‐related complications and interventions during decannulation and post‐decannulation periods. [file AOR-50-440-s005.docx]

**TABLE S5** Cannulation-related complications and interventions during decannulation and post-decannulation periods

|  | **Total**  **(n=384)** | **Percutaneous cannulation**  **(n=181)** | | | | **Surgical cannulation**  **(n=203)** | |
| --- | --- | --- | --- | --- | --- | --- | --- |
|  |  | **Decannulation**  **(n=117)** | | | | **Decannulation**  **(n=133)** | |
|  |  | **Percutan. decannul.** | **Surgical decannul.** | ***p*** | **All ^a^** | **Surgical decannul.** | ***p*** |
| **Decannulation** | 250 (65.1) | 66 (56.4) | 51 (43.6) | 0.02 | 117 (64.6) | 133 (65.5) | 0.86 |
| Cannulation-site bleeding ^b^ | 13 (5.2) | 7 (10.6) | 2 (3.9) | 0.30 | 9 (7.7) | 4 (3.0) | 0.10 |
| **Intervention** |  |  |  |  |  |  |  |
| Thrombectomy | 36 (14.4) | 4 (6.1) | 10 (19.6) | 0.03 | 14 (12.0) | 22 (16.8) | 0.30 |
| Vascular surgery ^c^ | 23 (9.2) | 3 (4.5) | 6 (11.8) | 0.18 | 9 (7.7) | 14 (10.5) | 0.44 |
| Conversion to surgical cutdown | 4 (1.6) | 4 (6.1) | n/a | n/a | n/a | n/a | n/a |
| **Post-decannulation** |  |  |  |  |  |  |  |
| Cannulation-site infection ^d^ | 57 (22.8) | 3 (4.5) | 8 (15.7) | 0.06 | 11 (9.4) | 46 (34.6) | <0.001 |
| Cannulation-site bleeding ^b^ | 25 (10.0) | 6 (9.1) | 7 (13.7) | 0.43 | 13 (11.1) | 12 (9.0) | 0.58 |
| Limb ischemia ^e^ | 10 (4.0) | 4 (6.1) | 0 (0) | 0.13 | 4 (3.4) | 6 (4.5) | 0.75 |
| Limb sensory-motor deficit | 9 (3.6) | 0 (0) | 4 (7.8) | 0.03 | 4 (3.4) | 5 (3.8) | 1.00 |
| Pseudoaneurysm | 8 (3.2) | 4 (6.1) | 2 (3.9) | 0.70 | 6 (5.1) | 2 (1.5) | 0.15 |
| **Intervention** |  |  |  |  |  |  |  |
| Cannulation-site revision ^f^ | 12 (4.8) | 4 (6.1) | 1 (2.0) | 0.39 | 5 (4.3) | 7 (5.3) | 0.72 |
| Vascular surgery (arterial site) ^c^ | 7 (2.8) | 4 (6.1) | 0 (0) | 0.13 | 4 (3.4) | 3 (2.3) | 0.58 |
| Thrombectomy | 4 (1.6) | 2 (3.0) | 0 (0) | 0.52 | 2 (1.7) | 2 (1.5) | 1.00 |
| Fasciotomy | 1 (0.4) | 0 (0 | 0 (0) | n/a | 0 (0) | 1 (0.8) | 1.00 |
| Amputation | 1 (0.4) | 0 (0) | 0 (0) | n/a | 0 (0) | 1 (0.8) | 1.00 |

Categorical variables are presented as n (%) and compared using χ² or Fisher’s exact test. Comparative analysis was performed between: 1) decannulation methods within the combined percutaneous group (percutaneous vs. surgical decannulation), and 2) the combined percutaneous group (column headed as ‘All’) and the decannulated patients in the surgical cannulation group.

^a^ Sum of percutaneous and surgically decannulated patients within the percutaneous cannulation group.

^b^ Major bleeding req. blood transfusion, cannula relocation, surgical cutdown, vascular repair.

^c^ Patch angioplasty, vascular suturing, desobliteration.

^d^ Local infection signs, positive culture of local pathogens, systemic septicemia indicators with local signs.

^e^ Clinical signs, sonographic evidence of limb ischemia, inadequate blood flow.

^f^ Surgical cutdown performed after completed decannulation.
