# Supplementary material for: Percutaneous Versus Surgical Cannulation for Femoro‐Femoral Venoarterial Extracorporeal Membrane Oxygenation: A Retrospective Cohort Study on Cannulation‐Related Complications
Source: Artif Organs. 2025 Nov 21;50(3):440–8. doi: 10.1111/aor.70061 (PMC13090744; doi:10.1111/aor.70061)
Supplement: Supplementary file 5 — Table S6: Patients with diagnoses classified as “Other.” [file AOR-50-440-s003.docx]

**TABLE S6** Patients with diagnoses classified as “Other”

| **Diagnoses** | **n (%)** |
| --- | --- |
| Primary cardiac arrhythmia | 20 (5.2) |
| Fulminant myocarditis | 13 (3.4) |
| Pulmonary embolism | 13 (3.4) |
| Drowning, severe hypothermia | 5 (1.3) |
| Acute heart transplant rejection | 3 (0.8) |
| Pulmonary arterial hypertension, severe ARDS | 3 (0.8) |
| Intoxication | 2 (0.5) |
| Peripartum cardiomyopathy | 2 (0.5) |
| Aortic annulus rupture after TAVI | 1 (0.3) |
| Takotsubo cardiomyopathy | 1 (0.3) |
| Pheochromocytoma crisis | 1 (0.3) |
| Traumatic cervical spinal cord injury | 1 (0.3) |
| Total | 65 (16.9) |

Variables are presented as n (%) of the whole study cohort (384 patients).

Abbreviations: ARDS, acute respiratory distress syndrome; TAVI, transcatheter aortic valve implantation.
