# Supplementary material for: Percutaneous Versus Surgical Cannulation for Femoro‐Femoral Venoarterial Extracorporeal Membrane Oxygenation: A Retrospective Cohort Study on Cannulation‐Related Complications
Source: Artif Organs. 2025 Nov 21;50(3):440–8. doi: 10.1111/aor.70061 (PMC13090744; doi:10.1111/aor.70061)
Supplement: Supplementary file 6 — Table S7: Nondecannulated patients with complications and interventions. [file AOR-50-440-s001.docx]

**TABLE S7** Non-decannulated patients with cannulation-related complications and interventions

|  | **Non-decannulated patients (n=134)** | **Non-decannulated patients**  **of total cohort**  **(n=384)** |
| --- | --- | --- |
| Cannulation-site bleeding | 32 (23.9) | 32 (8.3) |
| Limb ischemia | 21 (15.7) | 21 (5.5) |
| Cannulation-site infection | 7 (5.2) | 7 (1.8) |
| **Intervention** |  |  |
| Cannulation-site revision | 19 (14.2) | 19 (5.0) |
| Vascular surgery | 13 (9.7) | 13 (3.4) |
| Fasciotomy | 7 (5.2) | 7 (1.8) |
| Thrombectomy | 4 (3.0) | 4 (1.0) |
| Amputation | 1 (0.7) | 1 (0.3) |

Variables are presented as n (%). Excluding non-decannulated patients would underreport complications and interventions. Full definitions are provided in Supporting Information Table S1.
